# Supplementary material for: Rapid-Response Vector Surveillance and Emergency Control During the Largest West Nile Virus Outbreak in Southern Spain
Source: Insects. 2025 Oct 29;16(11):1100. doi: 10.3390/insects16111100 (PMC12653710; doi:10.3390/insects16111100)
Supplement: Supplementary file 1 [file insects-16-01100-s001.zip › Figure S1–S7 and Table S1.pdf]

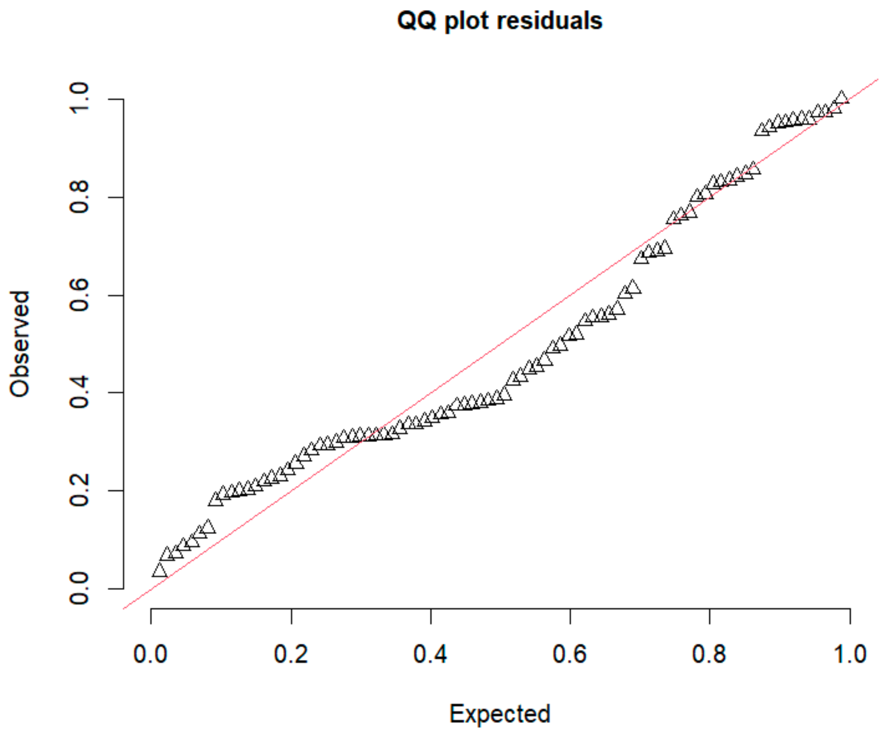

Figure S1

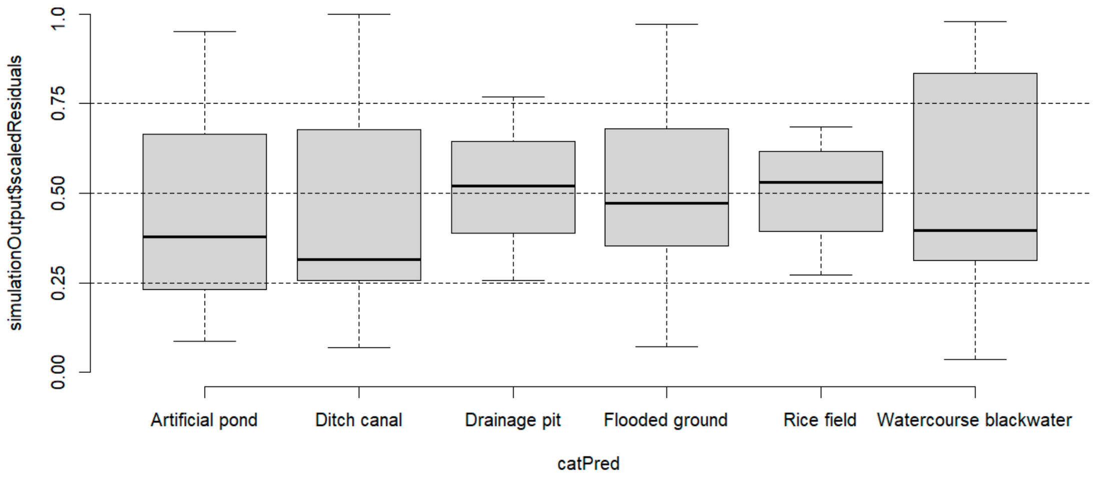

Figure S2

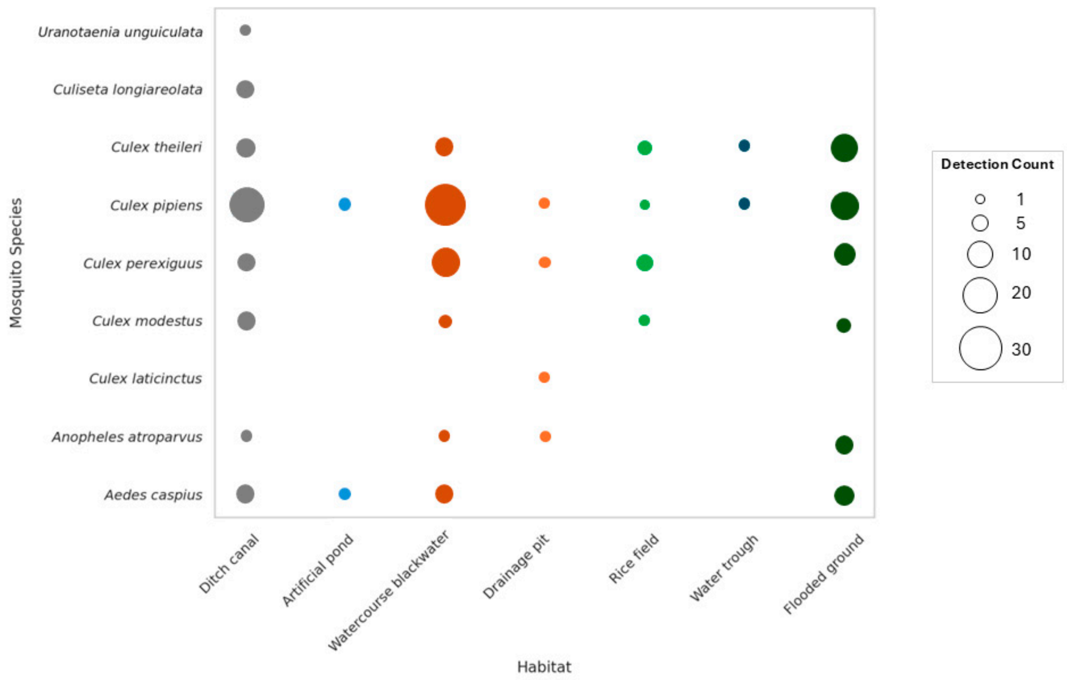
Uranotaenia unguiculata
Culiseta longiareolata
Culex theileri
Culex pipiens
Culex perexiguus
Culex modestus
Culex laticinctus
Anopheles atroparvus
Aedes caspius
Mosquito Species
Ditch canal
Artificial pond
Watercourse blackwater
Drainage pit
Rice field
Water trough
Flooded ground
Habitat
Detection Count
1
5
10
20
30

Figure S3.

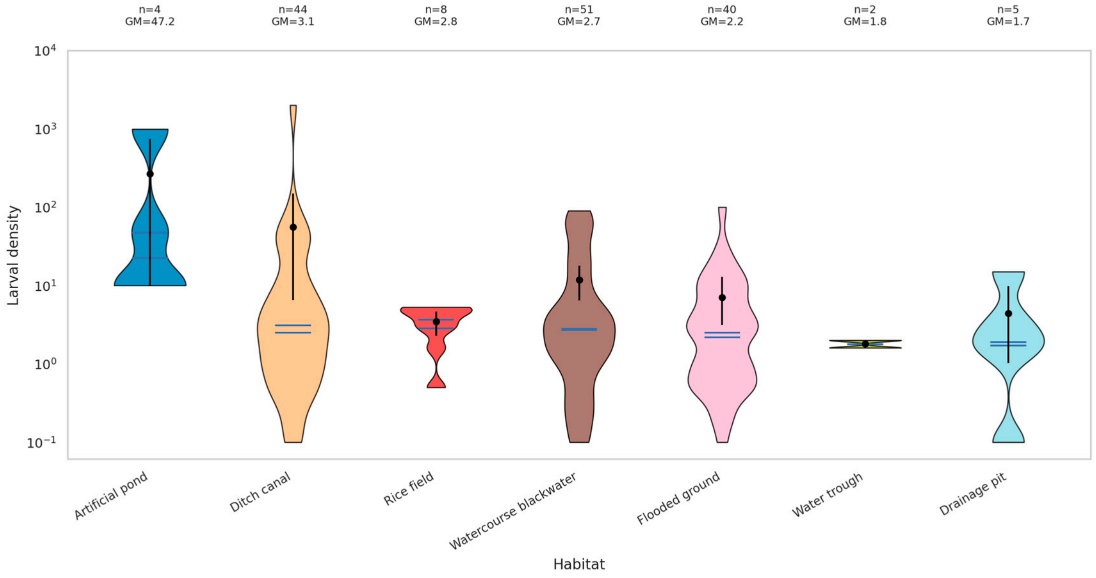
n=4
GM=47.2
n=44
GM=3.1
n=8
GM=2.8
n=51
GM=2.7
n=40
GM=2.2
n=2
GM=1.8
n=5
GM=1.7
10^4
10^3
10^2
10^1
10^0
10^{-1}
Larval density
Artificial pond
Ditch canal
Rice field
Watercourse blackwater
Flooded ground
Water trough
Drainage pit
Habitat

Figure S4

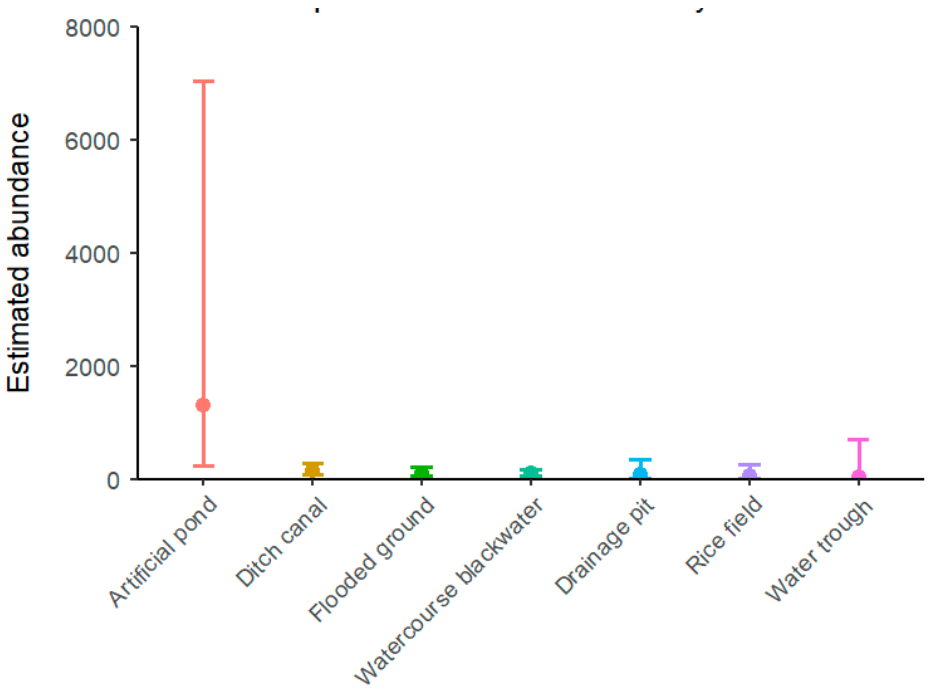

Figure S5

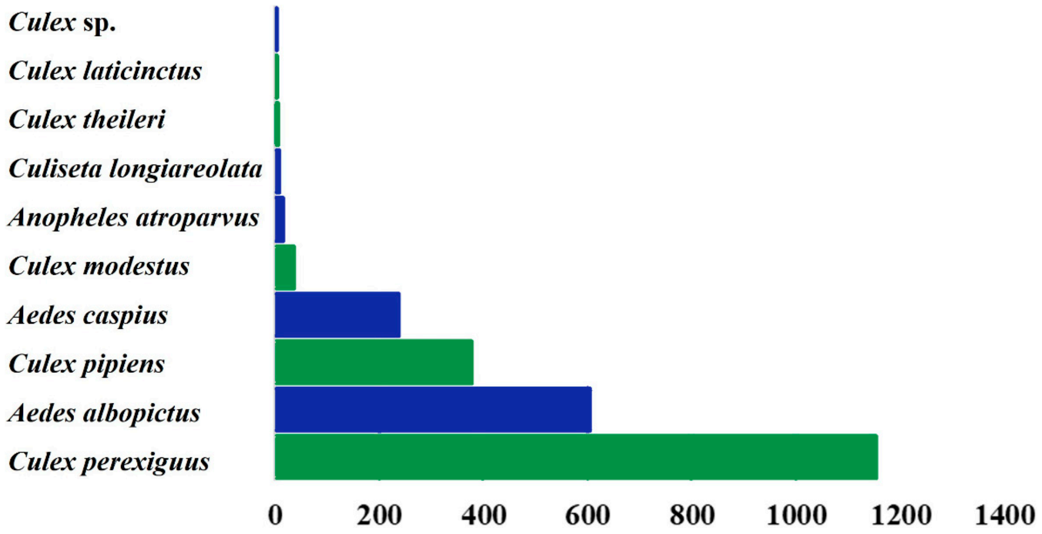

Figure S6

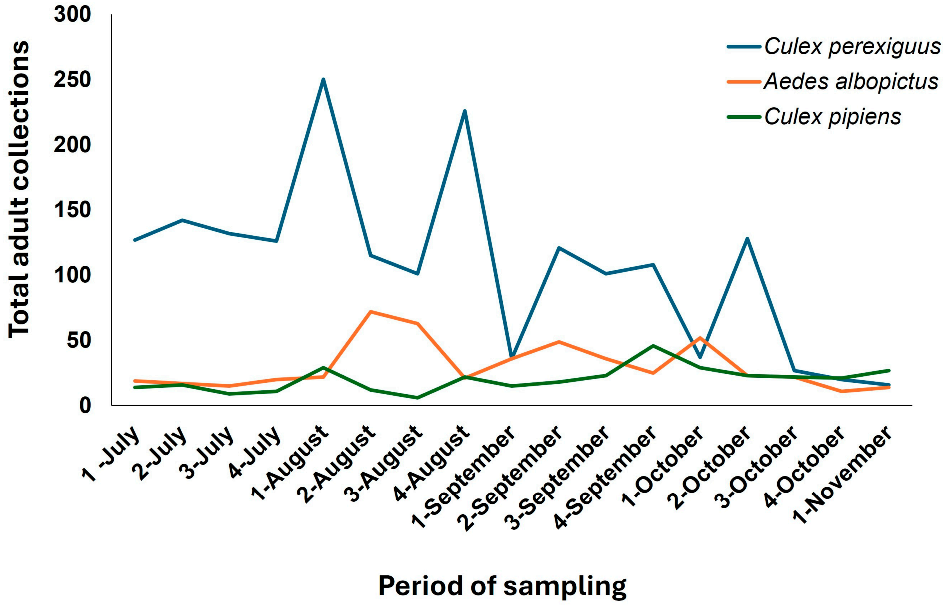

Total adult collections
300
250
200
150
100
50
0
Culex perexiguus
Aedes albopictus
Culex pipiens
1 -July
2-July
3-July
4-July
1-August
2-August
3-August
4-August
1-September
2-September
3-September
4-September
1-October
2-October
3-October
4-October
1-November
Period of sampling

Figure S7

Table S1

Species	Habitat	Municipality	Accesion nª
Cx. perexiguus	Rice field	UTRERA	-------------
Cx. perexiguus	Water course	DOS HERMANAS	-------------
Cx. perexiguus	Water course	DOS HERMANS	-------------
Cx. perexiguus	Water course	DOS HERMANAS	LC883883
Cx. perexiguus	Water course	DOS HERMANAS	LC883885
Cx. perexiguus	Water course	UTRERA	LC883887
Cx. perexiguus	Water course	DOS HERMANAS	LC883888
Cx. perexiguus	Rice field	DOS HERMANAS	LC883889
Cx. perexiguus	Water course	DOS HERMANAS	LC883882
Cx. perexiguus	Waterlogging	UTRERA	-------------
Cx. perexiguus	Waterlogging	DOS HERMANAS	LC883884
Cx. perexiguus	Water body	DOS HERMANAS	LC883886
Cx. perexiguus	Waterlogging	DOS HERMANAS	-------------
Cx. perexiguus	Water course	DOS HERMANAS	-------------
