# Supplementary material for: Rapid-Response Vector Surveillance and Emergency Control During the Largest West Nile Virus Outbreak in Southern Spain
Source: Insects. 2025 Oct 29;16(11):1100. doi: 10.3390/insects16111100 (PMC12653710; doi:10.3390/insects16111100)
Supplement: Supplementary file 1 [file insects-16-01100-s001.zip › Text_S1 and S2.pdf]

TEXT S1. Further methodological details and supporting data related to the phylogenetic analyses

The alignment included 24 sequences with 658 nucleotide positions, of which 64 were parsimony-informative sites, 58 singleton sites, and 536 constant sites. Model selection was conducted with ModelFinder [1] integrated in IQ-TREE, evaluating 286 models under the Akaike Information Criterion (AIC). The best-fit model selected was GTR+F+R4, corresponding to the general time-reversible model with empirical base frequencies (+F) and among-site rate heterogeneity modeled using the FreeRate approach with four rate categories (+R4). The estimated base frequencies were A = 0.292, C = 0.159, G = 0.152, and T = 0.396, and the final log-likelihood value was –1591.113. Branch support was assessed with 1,000 ultrafast bootstrap replicates (UFBoot) [2] and 1,000 SH-like approximate likelihood ratio tests (SH-aLRT). Branches with UFBoot values ≥ 95% and SH-aLRT values ≥ 80% were strongly supported. All sequences passed the chi-squared test for compositional homogeneity (p > 0.05), and identical sequences were retained and collapsed automatically in the analysis.

References:

1. Kalyaanamoorthy, S.; Minh, B.Q.; Wong, T.K.F.; Von Haeseler, A.; Jermiin, L.S. ModelFinder: Fast Model Selection for Accurate Phylogenetic Estimates. *Nat. Methods* **2017**, *14*, 587–589, doi:10.1038/NMETH.4285,.
2. Hoang, D.T.; Chernomor, O.; Von Haeseler, A.; Minh, B.Q.; Vinh, L.S. UFBoot2: Improving the Ultrafast Bootstrap Approximation. *Mol. Biol. Evol.* **2018**, *35*, 518–522, doi:10.1093/MOLBEV/MSX281.

TEXT S2: Residual Diagnostics for the Negative Binomial GLMM

To check for underlying structures not accounted by the fitted model, we visually inspected the overall distribution of simulated residuals (N = 500) compared to the expected uniform distribution under the negative binomial GLMM using a QQ plot from the DHARMa package. Additional tests were performed to statistically evaluate deviations of residuals from the expected uniform distribution and expected dispersion under the fitted model. The QQ plot (Fig. S1) and the Kolmogorov-Smirnoff test for overall uniformity showed no significant deviation from the expected distribution (asymptotic one-sample KS test: $D = 0.11637$; $p = 0.195$). The dispersion test neither showed overdispersion of residuals (dispersion = 5.6788; $p = 0.08$). Finally, we checked for differences in the distribution of the simulated residuals among habitat categories and in relation to the expected uniform distribution using a boxplot of residuals by category (Fig. 2), uniformity tests within-categories, and the Levene's test for homogeneity of variance, using the DHARMa

package. We did not find significant deviations from the expected uniformity within categories (all exact one-sample Kolmogorov-Smirnov tests: $p > 0.1$) neither between categories (Leven's test: $F = 0.553$; df = 5, 80; $p = 0.736$).
